# Supplementary material for: Zeolitic Imidazolate Framework-8 (ZIF-8) as a Drug Delivery Vehicle for the Transport and Release of Telomerase Inhibitor BIBR 1532
Source: Nanomaterials (Basel). 2023 May 31;13(11):1779. doi: 10.3390/nano13111779 (PMC10254680; doi:10.3390/nano13111779)
Supplement: Supplementary file 1 [file nanomaterials-13-01779-s001.zip › nanomaterials-2417444-supplementary.pdf]

Supporting Information

Zeolitic Imidazolate Framework-8 (ZIF-8) as a Drug Delivery Vehicle for the Transport and Release of Telomerase Inhibitor BIBR 1532

Shunyu Zhang [1,2,†], Jinxia Li [2,†], Liang Yan [2], Yue You [2], Feng Zhao [2,*], Jixing Cheng [1], Limin Yang [2], Yanqi Sun [3], Qingchao Chang [2], Ru Liu [2] and Yunhui Li [1,*]

1 Key Laboratory of Environmental Medicine Engineering of Ministry of Education, School of Public Health, Southeast University, Nanjing 210000, China; syzhang@ihep.ac.cn (S.Z.); chengjx0210@163.com (J.C.)
2 CAS Key Lab for Biomedical Effects of Nanomaterials and Nanosafety, Institute of High Energy Physics, Chinese Academy of Sciences, Beijing 100049, China; lijinxia@ihep.ac.cn (J.L.); yanliang@ihep.ac.cn (L.Y.); youyue@ihep.ac.cn (Y.Y.); yanglm@ihep.ac.cn (L.Y.); changqc@ihep.ac.cn (Q.C.); liuru@ihep.ac.cn (R.L.)
3 Department of Prevention and Health Care, Rizhao 276800, China; tangdou888@163.com (Y.S.)
* Correspondence: zhaof@ihep.ac.cn (F.Z.); yhli@seu.edu.cn (Y.L.); Tel.: +86-138-113-07996 (F.Z.); +86-135-125-38992 (Y.L.);
† These authors contributed equally to this work.

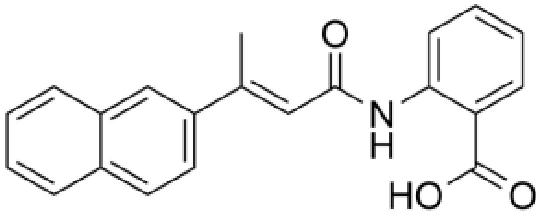

Figure S1. The structure of BIBR 1532.

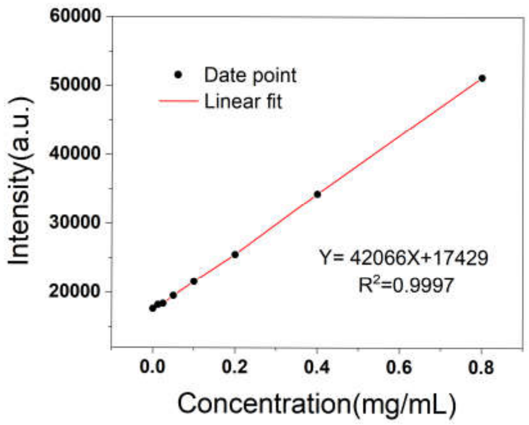

Figure S2. Plot of the standard curve. Fluorescence intensity values were plotted as functions of 6-AF@BIBR 1532 concentrations.

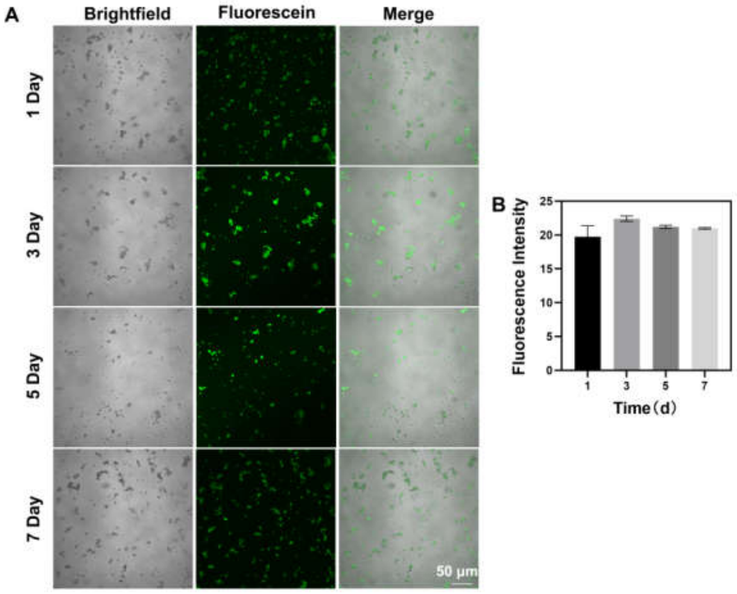

Figure S3. Fluorescence stability of fluorescein@ZIF-8. (**A**) Fluorescence signals of fluorescein@ZIF-8 detected at 1, 3, 5 and 7 days. (**B**) Quantitative analysis of fluorescence intensity using Image J.

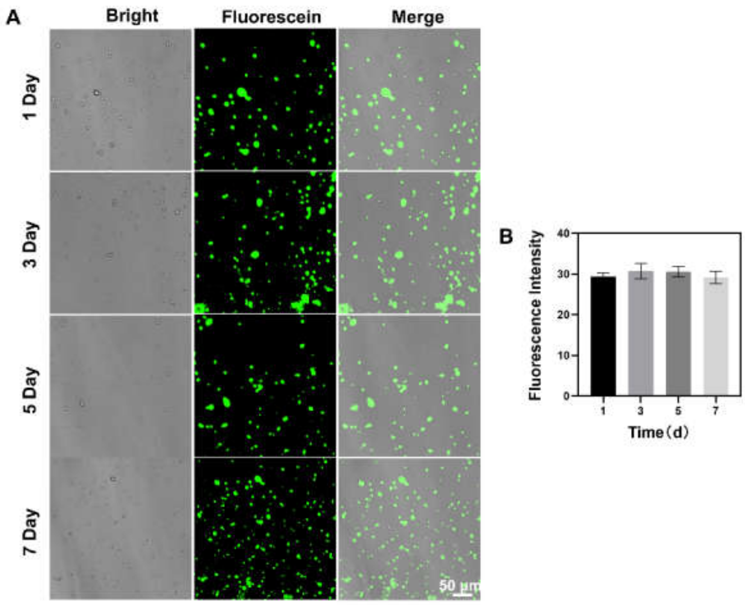

Figure S4. Fluorescence stability of 6-AF@BIBR 1532@ZIF-8. (**A**) Fluorescence signals of 6-AF@BIBR 1532@ZIF-8 detected at 1, 3, 5 and 7 days. (**B**) Quantitative analysis of fluorescence intensity using Image J.

Table S1. A summary of the similarities and differences between our present study and others' study ([78])

Similarities	Differences
**Starting point:**<br>BIBR 1532 has a hydrophobic structure and is insoluble in water;<br><br>**The role of the carrier:**<br>Acid-sensitive chemical bond releasing drugs;<br>The carrier allowed lysosomal escape of the drugs;	**Key observations:**<br>Our: the release of BIBR 1532 from ZIF-8; efficacy comparison of BIBR 1532 with or without ZIF-8 as a drug vector in aspect of proliferation, cell cycle and cellular senescence of tumor cells;<br>Others': the release of BIBR 1532 from PEG layer; the effect of telomerase inhibition by BIBR 1532 on DOX resistance reversal;<br><br>**Drug release：**<br>Our: pH-sensitive ZIF-8 helps release BIBR 1532;<br>Others': redox-sensitive PEG layer actualizes BIBR 1532 release;<br><br>**Functions of BIBR 1532：**<br>Our: BIBR 1532 inhibits tumor cell proliferation, induces cell cycle arrest and promote cellular senescence of tumor cells;<br>Others': BIBR 1532 weakens mitochondria protection and enhances ROS production and meanwhile promotes drug resistance tumor cell apoptosis;<br><br>**The significance of the experiment:**<br>Our: Improve the bioavailability of BIBR 1532 and enhance its biological effects;<br>Others': Stable loading of BIBR 1532 to build a telomerase-termination nanoplatform for DOX resistance reversal;
